# The impact of pet ownership on healthcare access and utilization among people with HIV

**DOI:** 10.1371/journal.pone.0292658

**Published:** 2023-11-01

**Authors:** Jennifer W. Applebaum, Shelby E. McDonald, Maya Widmeyer, Humberto E. Fabelo, Robert L. Cook

**Affiliations:** 1 Department of Environmental & Global Health, College of Public Health and Health Professions, University of Florida, Gainesville, Florida, United States of America; 2 Community Research and Evaluation, Denver Zoological Foundation, Denver, Colorado, United States of America; 3 Unconditional Love, Inc., Melbourne, Florida, United States of America; 4 School of Social Work, Virginia Commonwealth University, Richmond, Virginia, United States of America; 5 Department of Epidemiology, College of Public Health and Health Professions, University of Florida, Gainesville, Florida, United Stated of America; FHI 360, ZAMBIA

## Abstract

Though bonds with pets can be health-promoting for people with HIV (PWH), recent studies indicate that owning pets may complicate healthcare access, especially for those with fewer economic resources, poorer social support, and a strong human-animal bond. In this study, we make a case for considering pets to be an important element of the social environment that can influence healthcare access and utilization among PWH. Pet-owning PWH (*n* = 204) were recruited at healthcare and community sites throughout Florida as part of a larger survey study (the “Florida Cohort”). We developed a 12-item index of pet-related barriers to healthcare, which was designed to assess whether the participants experienced or anticipated any barriers to accessing and/or utilizing timely healthcare or health-related services due to pet caregiving or concerns about pet welfare. We estimated a series of regression models (negative binomial, logistic regression) to assess the effects of comfort from companion animals, human social support, healthcare needs, and sociodemographic characteristics on 1) the total number of pet-related healthcare barriers endorsed, 2) previously experienced pet-related healthcare barriers, and 3) anticipated pet-related healthcare barriers. Thirty-six percent of the sample reported at least one experienced or anticipated pet-related barrier to their healthcare; 17% reported previous healthcare barriers and 31% anticipated future healthcare barriers. Greater comfort from companion animals, greater healthcare needs, and poorer social support were associated with a greater probability of experiencing or anticipating any pet-related healthcare barriers. Those who identified racially as Black were less likely to anticipate future healthcare barriers than those who were White. Income was associated with pet-related healthcare barriers in all models. Given the importance of health maintenance for PWH and previous research suggesting pets may be an important emotional support for this population, social safety net programs and community partnerships that support multispecies families are strongly recommended.

## Introduction

Access to timely, high-quality healthcare is pertinent to maintaining one’s health, especially among those managing chronic diseases such as HIV. Social context is known to influence healthcare access and utilization. Behavioral models of healthcare utilization often include social determinants such as socioeconomic resources, community environment, and provider-related factors [1, 2], and these models have been expanded to recognize systemic and psychosocial factors as additional determinants of healthcare access and utilization [3–6].

Ryvicker’s [7] behavioral-ecological framework for healthcare access and navigation proposes that several factors lie between potential access to healthcare (i.e., services available to a patient) and realized access to care (i.e., services received). The behavioral-ecological framework builds upon the Andersen behavioral model of care access and utilization, which posits that predisposing (e.g., sociodemographic characteristics, health beliefs), enabling (e.g., access to resources), and need factors (e.g., health status) determine health service use [4, 8]. Ryvicker’s model builds upon Andersen’s to include the social environment, neighborhood and built environment characteristics, and the healthcare environment [7]. They further argue that features of the environments in which the person is embedded can shape the navigation process, thus impacting healthcare decision-making, and ultimately, realized access [7].

Though pet ownership is commonly thought to impact health, it has rarely been considered a facilitator and/or barrier in healthcare access and utilization. In this study, we draw on the limited existing literature on pet ownership and healthcare decision-making as well as the behavioral-ecological framework of healthcare access and navigation [7] to make a case for considering pets to be an important element of the social environment that can influence healthcare access and utilization among people with HIV (PWH).

### Healthcare utilization and access among PWH

Today, PWH have better outcomes and prognosis when diagnosed with the disease than in earlier decades. Advances in disease treatment have resulted in the evolution of HIV from a terminal illness to a chronic illness [9]. Standard treatment achieves results of viral suppression for most patients which allows the ability to maintain daily activities and normal routines with a typical lifespan. However, patients must adhere to their antiretroviral therapy (ART) medications and regular medical appointments. Unlike other chronic illnesses, regular medical appointments involve more frequent routine screening of standard labs, annual vaccination, and routine atypical screening, in addition to more frequent provider visits compared to a patient without HIV. Routine screening for a patient with HIV will include HIV viral load, CD4 count, hematology studies, chemistry studies, and urine analysis [10]. Atypical regular screenings include screening of sexually transmitted diseases, opportunistic infections, and cancers. Patients are required to see their HIV provider and have a HIV viral load assessment and CD4 count every three to six months [10]. As the PWH population ages, patients will experience the typical comorbidities of aging, as well as the long-term effects of HIV in the body and the long-term effects of damage caused by ART medications. The minimum additional specialists that will eventually be brought into their center of care are in the areas of cardiology, neurology and nephrology. It is estimated that HIV related medical cost for a person with HIV will be between $420,285 and $1,079,999 during their lifetime [11].

Strict adherence to one’s treatment regimen and regular healthcare can be a challenge for people who face structural barriers to health maintenance [12]. PWH are a population in which barriers to access to healthcare are amplified due to the structural barriers in place for populations that are over-represented among PWH: PWH in the U.S., compared to the overall U.S. population, are disproportionately low-income, identify as a sexual and gender minority, and Black or Hispanic/Latine [13]. In particular, racial and ethnic disparities in new HIV diagnoses are notable: Black Americans and Hispanic/Latine Americans account for disproportionately high rates of new diagnoses in recent years [14]. Inequalities in access to overall healthcare and HIV prevention, and once diagnosed, HIV care, are salient factors contributing to HIV management and overall health in this population [14, 15]. PWH are also subject to social stigma, which can extend to the healthcare setting and thus become a barrier to access and utilization [16–19].

Because of the many structural and individual challenges associated with the social positions of PWH, access to healthcare must be conceptualized beyond geographic proximity and health insurance status in order to optimize care for PWH [20]. In addition to economic and stigma-related barriers, Asghari et al. [20] highlight caregiving responsibilities as a barrier to healthcare access for PWH: patients who had dependent children could not as easily attend their healthcare appointments because they were unable to bring their children with them. Social support and/or access to childcare is thus likely pertinent for PWH with caregiving responsibilities and may moderate the effect of income on healthcare access and utilization: hypothetically, those with few economic resources but a robust and supportive social network may be able to rely on their network for care of their dependents, including pets [21]. In this way, caring for a pet, which mirrors many of the responsibilities and demands of caring for children, could be considered an individual factor that prevents the utilization of necessary healthcare, particularly in the context of other limiting factors, such as scarce socioeconomic resources. However, pet ownership may also be conceptualized as an enabling factor for healthcare utilization, as pets are known to provide emotional support and motivation to maintain health, particularly among those with chronic diseases, such as HIV [22–24].

### Relationships with pets among PWH

Previous research suggests that pets can motivate caregivers with HIV to maintain their health in order to be physically able and available to care for their pets [22–24]. Other research has consistently shown that pets can offer nonjudgmental emotional support and companionship to PWH, particularly for those who are otherwise socially isolated [22–28]. The emotional comfort PWH can derive from a pet is thought to be beneficial to health and well-being. For example, in qualitative studies, pet ownership buffered PWH from social isolation and stigma by providing social support and a caregiving role, thus providing motivation to carry on when life is otherwise challenging [22, 29]. For example, Kabel and colleagues reported that pets, particularly dogs, provided emotional support and unconditional love for women with HIV who experienced social stigma and marginalization in the wake of their diagnoses [27]. Pet caretaking can also provide a meaningful social role that contributes to stress reduction and thus promotes effective self-management of HIV in daily caregiving tasks and the provision of reciprocal love and affection [24]. In some cases, support from pets may complement the beneficial health effects of support from one’s social network [26]. However, there is some indication that those who have stronger attachment bonds to their pets may seek out social support from pets to replace poor support from people. For example, Hutton found that, though PWH who lived with a pet reported greater well-being and fewer experiences of poor social interactions than those who did not have a pet, among the pet owners, those with higher attachment to their pets had poorer well-being and greater experiences of poor social interactions than those with lower attachment [25].

Though pet ownership is often thought of as beneficial for PWH, it is not without risks to this population. For example, pet caretaking can compromise health for people with immunodeficiency, such as the risk of contracting toxoplasmosis from cat feces [29]. Furthermore, the expense of caring for a pet may compete for household resources, thus complicating access to healthcare and health-promoting resources [30–33].

### Pet ownership and healthcare access and utilization

Pets are known to influence healthcare decisions and access, particularly in the case of hospitalization or incapacitation due to acute public health emergencies like COVID-19 [33, 34]. For example, in the case of the COVID-19 pandemic, some pet owners indicated that they would not seek healthcare if they became ill because they were concerned about what might happen to their pet if they were to become hospitalized, which was especially salient for those who lacked support from their social network, as well as those who were economically insecure [33]. A salient concern highlighted across studies was the availability (or lack thereof) of personal network members to provide contingency care for pets in the case of illness or hospitalization, further highlighting the pertinence of social support above and beyond resource constraints [21, 32–34]. Furthermore, prior to the COVID-19 pandemic, one study found that half of the pet owners in their sample would delay hospitalization due to pet caregiving responsibilities, and those with poor social support from people were most at-risk [35]. In the case of those with severe resource constraints, such as individuals experiencing homelessness, pet ownership may completely prevent them from accessing healthcare because they cannot bring the animals with them to health clinics and don’t have space or resources for alternative care [36]. This problem has not been considered previously among PWH, a population that both requires regular engagement with the healthcare system and is disproportionately representative of low-resourced and marginalized social groups [13, 37, 38].

### The current study

In this study, we draw upon the literature on sociodemographic disparities in access to and utilization of healthcare, pet ownership and the human-animal bond among people with HIV, and broader sociodemographic health disparities to examine whether pet ownership is a potential barrier to healthcare access and utilization among PWH. If pets are indeed beneficial to health and well-being among PWH (as previous research has suggested), but pet caregiving can also exacerbate economic and social strain for these same individuals, it is therefore important to understand how policy and practice may aim to mitigate some of the healthcare barriers presented by pet ownership in this population. We also draw from the behavioral-ecological model of healthcare access and navigation [7] to argue that pet ownership should be considered a feature of the social environment that can act as both a facilitator and barrier to realized access to healthcare. Specifically, in this study, we test whether social support from people, number of pets in the home, type of pets in the home, the extent of comfort derived from one’s pet, household income, race, Hispanic ethnicity, and variation in healthcare needs are associated with the extent to which PWH experience and/or anticipate experiencing pet-related barriers to their healthcare. We hypothesize that greater comfort derived from companion animals, lower levels of social support, lower income, and greater healthcare needs will be associated with more pet-related barriers to healthcare, above and beyond the effects of race, ethnicity, number of pets, and types of pets in the home. Additionally, based on prior research showing the importance of social support, income, and bonds with pets on access and utilization of healthcare [33, 35, 39, 40], we test exploratory hypotheses that social support will moderate the effects of both income and comfort from pets on pet-related barriers to healthcare.

## Methods

Data were from Wave 3 of the Florida Cohort, a longitudinal survey of PWH in Florida, run by the Southern HIV and Alcohol Research Consortium (SHARC). The goal of the study is to “assess how individual, clinic, and community level factors influence healthcare accessibility and utilization and HIV clinical outcomes across the state of Florida” [41]. Methodology from Wave 2 of the Florida Cohort is described in Ibanez et al. [42]. Wave 3 had a similar approach and goals to Wave 2, with some differences in recruitment locations and new measures. Participant recruitment was via HIV care providers throughout Florida, patient registries, participant referrals, and remotely via digital and paper advertising. Data collection was conducted from 2021–2023. Florida Cohort participants were eligible for several survey modules that cover general health, health care utilization, behavioral and social factors, substance use, and mental health. If the participants identified themselves as pet owners, they were eligible to complete a module that is specific to interactions with pets. The pet module, which was available in English and Spanish languages, included a variety of human-animal interaction measures that are intended to assess positive, negative, and neutral aspects of pet ownership among PWH. Participants gave written consent for participation and were compensated for each module they completed. The authors did not have access to identifying information of the participants. The study was approved by the University of Florida’s Internal Review Board, protocol number IRB201801680.

### Sample characteristics

As of August 2023, 735 PWH completed the Florida Cohort core module. Of the 735 participants, 43% (*n* = 317) endorsed current pet ownership, and 30% (*n* = 219) completed the pet module. Only observations with full information on each variable included in the analytic models were retained, which resulted in removing 15 observations for a sample size of 204. Descriptive information for the sample can be found in Table 1.

**Table 1:** Characteristics of 204 persons with HIV who completed the Florida Cohort pet module.

Variable	Proportion or Mean (SD) (range)
*Race*	
White	66%
Black	28%
Other race	6%
Hispanic	18%
*Income*	
Less than $10,000 per year	25%
$10,000–29,999 per year	38%
$30,000–49,999 per year	23%
$50,000 and above per year	14%
*Education*	
Less than high school	14%
High school graduate	32%
College and above	54%
*Gender*	
Cisgender male	57%
Cisgender female	40%
Transgender man	0%
Transgender woman	2%
Other gender	1%
Age	49 (12.5) (21–75 years)
Comfort from Companion Animals Scale	42 (8.2) (12–48)
Social Support Scale	45 (12.5) (12–60)
Dog owner	70%
Cat owner	42%
Other pet type owner	39%
Number of pets	2 (1.7) (1–10)
Taking antiretroviral (ART) medication	93%
Missed any HIV healthcare appointments in past 12 mos	23%
Only seeing HIV provider for healthcare in past 12 mos	18%
Hospitalized overnight at least once in past 12 mo	26%
Years since HIV diagnosis	19 (11.3) (1–42)

### Measures

#### Pet-related barriers to healthcare

The outcome measure in this study is an index of pet-related barriers to healthcare that was developed for this survey module. We developed the index by drawing on previous research that focused on pet caregiving responsibilities and concerns about pet welfare as potential or actual barriers to healthcare access and utilization [33, 35, 36]. We also consulted with HIV care providers regarding the validity of the constructs being measured based on their experiences with patients in the field. The index includes a series of 12 questions designed to assess whether the participant experienced or anticipated any barriers to accessing and/or utilizing timely health care or health-related services due to pet caregiving or concerns about pet welfare (see Table 2). The index was coded in multiple ways for analytic purposes. For the negative binomial regression, the index was treated as a count outcome with values 0–12, representing the total number of items endorsed (Cronbach’s alpha = 0.88, McDonald’s omega = 0.86). For the logistic regression models, the index was recoded into two binary outcomes. The first, measuring whether participants had ever experienced previous pet-related barriers to healthcare (Cronbach’s alpha = 0.88, McDonald’s omega = 0.86), was coded 1 if a participant endorsed any of items #1–8 in the index (or coded 0 otherwise). The second, measuring whether participants anticipated experiencing future pet-related barriers to healthcare (Cronbach’s alpha = 0.88, McDonald’s omega = 0.88), was coded 1 if a participant endorsed any of the items #9–12 in the index (or coded 0 otherwise).

**Table 2:** Items included in the pet-related barriers to healthcare index.

#	Item
	*Have you ever*:
1	Delayed seeking health services because you were worried about your pets?
2	Delayed seeking health services because you had to take care of your pets?
3	Not received health services because you were worried about your pets?
4	Not received health services because you had to take care of your pets?
5	Left in-patient health services because you were worried about your pets?
6	Left in-patient health services because you had to take care of your pets?
7	Have your pets ever impacted your ability to pay for medication?
8	Have your pets ever impacted your ability to pay for health services other than medication?
	*Would you*:
9	Delay seeking health services if it meant your absence would cause stress for your pets?
10	Delay seeking health services if it meant you could not care for your pets?
11	Miss health services if it meant your absence would cause stress for your pets?
12	Miss health services if it meant you could not care for your pets?

#### Comfort from companion animals

The Comfort from Companion Animals Scale (CCAS) is a 12-item measure designed to assess the extent to which pet owners receive comfort from their companion animal(s) [43]. Participants completed the 12-item scale, which included items such as “Having a pet gives me something to care for,” and “I get comfort from touching my pet.” Response options were on a 4-point Likert scale of agreement, ranging from strongly disagree (1) to strongly agree (4). Responses for each item were summed and scores ranged from 12 (low comfort) to 48 (high comfort). Both the Cronbach’s alpha (0.98) and McDonald’s omega (0.98) coefficients or the scale both reflected good reliability. The CCAS has previously been used in research with PWH [26].

#### Pet type and number

Participants were asked, “What type of pets do you have currently? (Check all that apply).” The options to select were dog, cat, bird, horse, fish, reptile, small mammal, and other. For analytic purposes, we recoded the pet type variables to indicators for (1) dog, (2) cat, and (3) all others. Participants were also asked how many pets or companion animals they currently had, to which they gave a numerical response.

#### Social support

Participants completed the 12-item Multidimensional Scale of Perceived Social Support [44], which assessed their subjective perception of support from their social network. The scale ranged from scores of 12 (low social support) to 60 (high social support). Response options were on a 5-point Likert scale, ranging from strongly disagree (1) to strongly agree (5) with a neutral mid-point choice or neither agree nor disagree (3). The scale, which had good reliability (Cronbach’s alpha = 0.95, McDonald’s omega = 0.95), included items such as “There is a special person who is around when I am in need,” and “My family really tries to help me.”

#### Income

Participants self-reported their yearly household income in nine groups from <$5k to $100k and above. Due to small group sizes in several of the income categories, we recoded the income groups to (1) less than $10,000 per year, (2) $10,000–29,999, (3) $30,000–49,999, and (4) $50,000 and above. In this study, income is an indicator of socioeconomic status, as well as an enabling or limiting factor based on healthcare access and utilization models [4, 7, 45]. Though socioeconomic status is often operationalized as income, education, and occupational status, due to data limitations, we only include income in these analyses.

#### Race

Participants self-identified their race as White, Black, Native American/American Indian/Indigenous, Pacific Islander, Asian, or other races. The three race groups in this study were White (1), Black (2), and other race(s) (3). Those who did not identify as White or Black were grouped into “other race(s)” due to small group sizes. This approach was employed for analytic purposes, as small group sizes are not compatible with the methodologies used in this paper. There are limitations to grouping racial and ethnic groups; for a discussion of the issues this approach can raise see [46].

#### Hispanic ethnicity

Participants were asked, “Do you consider yourself to be of Hispanic, Latino/a, or Spanish origin?” Those who endorsed Hispanic ethnicity were coded 1, and those who did not were coded 0. The choice to retain race and Hispanic ethnicity as separate variables in this study was driven primarily by our analytic approach, as well as the acknowledgment that Hispanic individuals can be of many different races and thus have different experiences related to race and racism. Additionally, we have chosen to use the term “Hispanic” here (instead of Latino/a/x/e) for consistency with the survey language.

#### Indicators of healthcare needs

ART: Participants were asked, “Are you currently taking HIV antiretroviral medications?” Response options were yes or no.

Missed appointments: Participants were asked, “Have you missed any scheduled HIV health care appointments in the past 12 months?” Response options were yes or no.

Other healthcare providers: Participants were asked, “Other than your HIV health care provider, have you seen any of the following doctors or healthcare providers in the past 12 months? (Check all that apply).” Response options were “A primary care doctor (different from your HIV provider),” “A women’s health specialist (gynecologist, OB/GYN),” “A psychiatrist, psychologist, or counselor,” “A dentist,” “Other,” and “I have not seen another healthcare provider.” The indicator for no other providers in the past 12 months was included in the analyses.

Hospitalized overnight: Participants were asked, “How many times have you been hospitalized overnight in the past 12 months?” Participants gave a numerical response and we binarized the variable to indicate whether they had been hospitalized at least once (1) or not at all (0).

Time since HIV diagnosis: Participants were asked, “What year did you first test positive for HIV? (Your best guess is OK).” Response options were given in four-digit years. We subtracted the year given from the year the survey was completed to indicate years since diagnosis.

### Analytic procedures

We first obtained univariate information for each of the 12 items on the pet-related barriers to healthcare index, the overall count of the number of items endorsed, the proportion of the sample that endorsed at least one item, and the proportions of the sample that endorsed (1) the eight questions that ask about previous experiences with pet-related barriers to healthcare, and (2) the four questions that ask about anticipated future pet-related barriers to healthcare. A chi-squared test was conducted to assess the association between previously experienced barriers and anticipated barriers. Next, we estimated a series of regression models to assess the effects of comfort from companion animals, social support, race, Hispanic ethnicity, income, pet type and number, and healthcare needs on experiences of pet-related barriers to healthcare. Because the data were overdispersed (LR *X*^2^ = 543.27, *p* = 0.000), a negative binomial regression (rather than Poisson) was estimated for the model predicting the number of healthcare barriers [47]. Logistic regression models were estimated for (1) the likelihood of endorsing at least one item related to previously experienced pet-related barriers to healthcare, and (2) the likelihood of endorsing at least one item related to anticipated future pet-related barriers to healthcare. Finally, we tested whether social support moderated the effects of (1) income and (2) comfort from companion animals on the likelihood of experiencing and/or anticipating pet-related barriers to healthcare by including interaction terms in the regression models.

## Results

**Fig 1:**
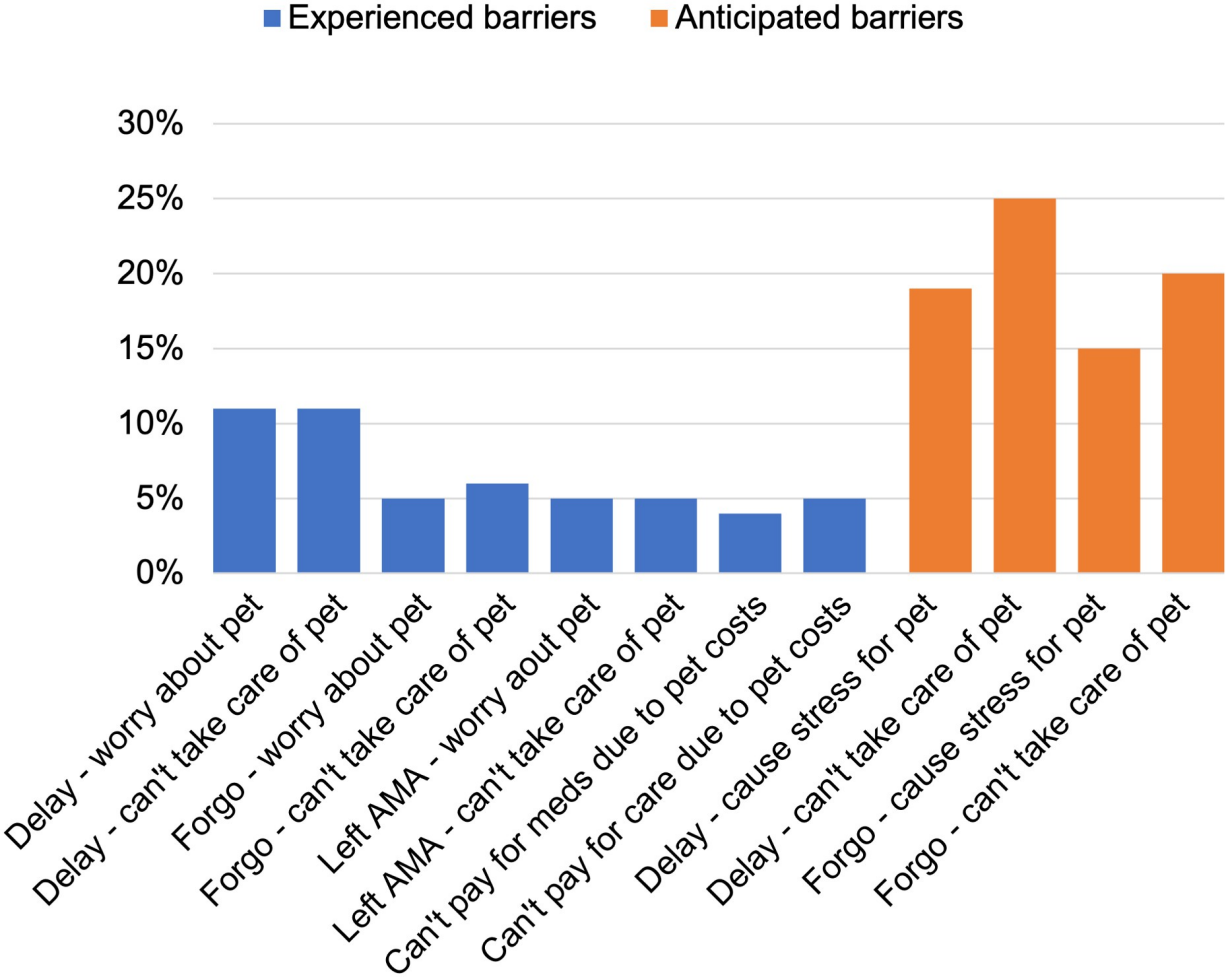
Relative frequencies of items endorsed in the pet-related barriers to healthcare index (*n* = 204).

Fig 1 displays the proportions of the pet module participants who endorsed each item in the pet-related barriers to healthcare index. Thirty-six percent of the sample endorsed at least one item on the index. The item that was endorsed most frequently was “Would you delay seeking health services if you could not care for your pet?” (25%), and the least frequently endorsed item was “Have your pets ever impacted your ability to pay for medication?” (4%). More participants endorsed items indicating anticipated healthcare barriers (31%) than the items indicating previously experienced healthcare barriers (17%). Endorsing at least one previously experienced barrier was significantly associated with endorsing at least one anticipated future barrier: 73% of those who reported previous barriers also endorsed anticipation of future barriers (*X*^2^(1) = 37.31, *p* = 0.000).

### Predicted number of pet-related barriers to healthcare

**Fig 2:**
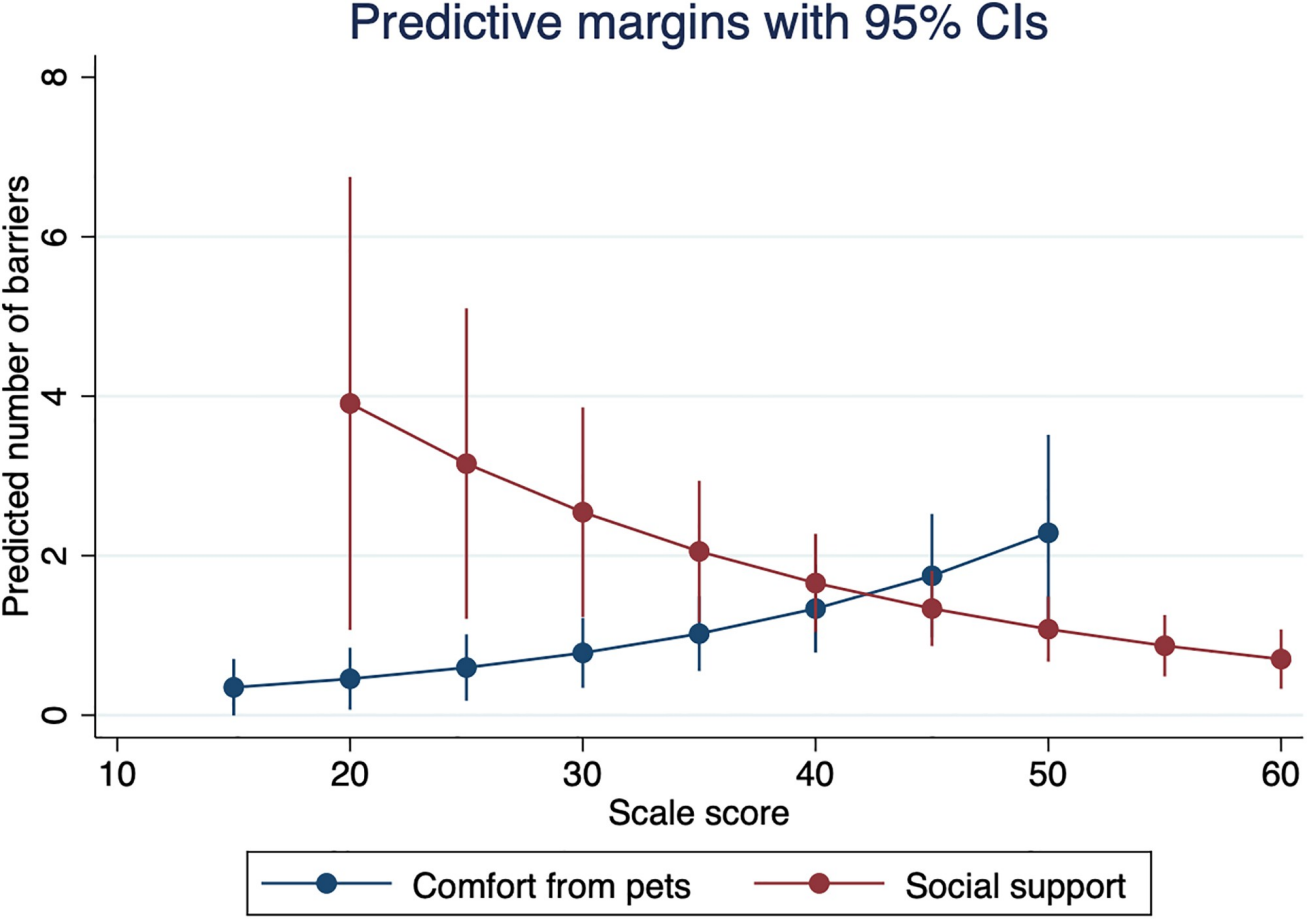
Predictive margins for the number of pet-related barriers to healthcare by comfort from companion animals and social support (*n* = 204). Adjusted for race, Hispanic ethnicity, income, pet type, number of pets, and healthcare needs.

Those who reported making $10,000–29,999 per year reported fewer total healthcare barriers, compared to those earning less than $10,000 per year (*IRR* = 0.38, *p* = 0.016), holding other variables constant. Those who reported greater comfort from companion animals reported greater healthcare barriers (*IRR* = 1.05, *p* = 0.009). Those who reported having more social support reported fewer total healthcare barriers (*IRR* = 0.95, *p* = 0.000) (see Fig 2). Participants who were taking ART endorsed a greater number of barriers than those who were not taking ART (*IRR* = 4.56, *p* = 0.045). No other variables in the model were significantly associated with the number of pet-related barriers to healthcare (see Table 3).

**Table 3:** Negative binomial model predicting incidence rate ratios (IRR) for pet-related barriers to healthcare (*n* = 204).

Variable	IRR (S.E.)	95% CI
Race (ref = White)		
Black	0.55 (0.19)	0.28–1.07
Other race	0.64 (0.46)	0.15–2.65
Hispanic	0.63 (0.26)	0.28–1.39
Income (ref = <$10k)		
$10k-29,999	0.38* (0.15)	0.18–0.82
$30k-49,999	1.14 (0.46)	0.52–2.51
$50k+	0.63 (0.29)	0.25–1.59
Comfort from Companion Animals Scale	1.06** (0.02)	1.02–1.09
Social Support Scale	0.96** (0.01)	0.93–0.98
Dog owner	1.36 (0.66)	0.52–3.52
Cat owner	0.89 (0.39)	0.37–2.12
Other pet type owner	1.18 (0.37)	0.65–2.17
Number of pets	1.20 (0.11)	0.99–1.45
Taking ART	4.56* (3.45)	1.03–20.06
Missed appt in past 12 mo	1.10 (0.44)	0.49–2.41
No other providers	0.53 (0.22)	0.23–1.20
Hospitalized overnight in past 12 mo	1.24 (0.50)	0.73–2.86
Years since diagnosis	1.00 (0.01)	0.98–1.03
Likelihood ratio *X*^2^(df)	37.18(17)**	

*p<0.05,

**p<0.01,

***p < .001

### Likelihood of having experienced previous pet-related barriers to healthcare

Income and social support were both significant in the model predicting the likelihood of having experienced previous pet-related barriers to healthcare. Specifically, compared to those making less than $10,000 per year, those who made $30,000–49,999 per year had 3.64 times the odds of previously experiencing healthcare barriers (*p* = 0.039). As social support increased, the odds of having experienced previous healthcare barriers decreased (OR = 0.94, *p* = 0.000). No other variables in the model were significantly associated with the likelihood of having experienced previous pet-related barriers to healthcare (see Table 4).

**Table 4:** Logistic regression models predicting odds ratios (OR) for previously experienced at least one pet-related barrier to healthcare (Model 1–2), and anticipation of at least one future pet-related barrier to healthcare (Models 3–4) (*n* = 204).

Variable	Previously experienced barriers				Anticipated future barriers			
	Model 1		Model 2		Model 3		Model 4	
	OR (S.E.)	95% CI	OR (S.E.)	95% CI	OR (S.E.)	95% CI	OR (S.E.)	95% CI
Race (ref = White)								
Black	0.85 (0.40)	0.33–2.16	0.86 (0.41)	0.34–2.17	0.27** (0.12)	0.11–0.66	0.22** (0.11)	0.09–0.57
Other race	0.96 (0.88)	0.16–5.83	1.02 (0.96)	0.16–6.42	0.39 (0.35)	0.07–2.23	0.48 (0.45)	0.08–2.97
Hispanic	0.43 (0.27)	0.12–1.51	0.50 (0.33)	0.14–1.79	0.53 (0.27)	0.19–1.44	0.67 (0.36)	0.23–1.93
Income (ref = <$10k)								
$10k-29,999	1.29 (0.72)	0.43–3.88	12.30 (22.00)	0.37–409.97	0.17*** (0.08)	0.07–0.42	23.88* (35.52)	1.01–563.66
$30k-49,999	3.64* (2.27)	1.07–12.37	46.09 (103.54)	0.56–3764.97	0.31* (0.16)	0.11–0.85	9.98 (20.05)	0.19–512.11
$50k+	2.33 (1.65)	0.58–9.35	810.58* (2681.08)	1.24–529958.7	0.39 (0.23)	0.13–1.21	1.08e+09 (9.56e+09)	33.30–3.25e+16
Comfort from Companion Animals Scale	1.02 (0.03)	0.97–1.08	1.02 (0.03)	0.97–1.08	1.06* (0.03)	1.01–1.11	1.06* (0.03)	1.00–1.12
Social Support Scale	0.94*** (0.02)	0.91–0.97	0.99 (0.03)	0.93–1.06	0.96** (0.01)	0.93–0.99	1.05 (0.03)	0.99–1.11
Dog owner	0.63 (0.36)	0.21–1.92	0.64 (0.37)	0.21–1.99	1.45 (0.75)	0.52–3.99	1.71 (0.96)	0.57–5.15
Cat owner	0.42 (0.25)	0.14–1.34	0.43 (0.26)	0.13–1.39	0.95 (0.49)	0.34–2.61	1.07 (0.61)	0.35–3.27
Other pet type owner	1.32 (0.54)	0.59–2.95	1.43 (0.60)	0.63–3.27	0.99 (0.36)	0.48–2.02	1.16 (0.46)	0.53–2.54
Number of pets	1.16 (0.14)	0.93–1.46	1.15 (0.14)	0.91–1.45	1.09 (0.13)	0.87–1.38	1.03 (0.13)	0.81–1.31
Taking ART	3.03 (3.37)	0.34–26.78	2.86 (3.03)	0.29–27.62	7.91 (8.86)	0.88–71.14	17.41 (26.55)	0.88–346.01
Missed appt in past 12 mo	1.13 (0.56)	0.43–2.98	1.28 (0.65)	0.47–3.48	1.18 (0.52)	0.49–2.81	1.59 (0.76)	0.63–4.05
No other providers	0.73 (0.40)	0.25–2.13	0.65 (0.37)	0.21–1.96	0.74 (0.35)	0.29–1.88	0.59 (0.31)	0.21–1.63
Hospitalized overnight in past 12 mo	1.01 (0.27)	0.60–1.70	0.95 (0.26)	0.56–1.62	1.28 (0.29)	0.82–2.00	1.20 (0.29)	0.75–1.94
Years since diagnosis	1.00 (0.02)	0.97–1.04	1.01 (0.02)	0.97–1.04	0.99 (0.16)	0.97–1.03	0.99 (0.02)	0.96–1.03
Income x Social Support Scale (ref = <$10k)								
$10k-29,999			0.94 (0.04)	0.87–1.03			0.88** (0.03)	0.82–0.96
$30k-49,999			0.94 (0.05)	0.85–1.03			0.92 (0.04)	0.84–1.00
$50k+			0.87 (0.07)	0.74–1.01			0.61* (0.12)	0.41–0.91
Likelihood ratio *X*^2^(df)	24.40(17)		29.12(20)		50.18(17)***		78.01*** (20)	

*p<0.05,

**p<0.01,

***p<0.001

### Likelihood of anticipating future pet-related barriers to healthcare

Race, income, social support, and comfort derived from pets were significantly associated with the likelihood of anticipating future pet-related barriers to healthcare. Compared to White participants, those who identified racially as Black had a 0.29 odds decrease in anticipating future healthcare barriers (*p* = 0.006). Compared to those who made less than $10,000 per year, those who made $10,000–29,999 per year had a 0.17 odds decrease in anticipating future healthcare barriers (*p* < 0.000), and those who made $30,000–49,999 per year had a 0.30 odds decrease (*p* = 0.020). For each unit increase in the Comfort from Companion Animals scale, the odds of anticipating future healthcare barriers increased by 1.06 (*p* = 0.023). For every unit increase in the social support scale, the odds of anticipating future healthcare barriers decreased by 0.96 (*p* = 0.006). No other variables in the model were significantly associated with the likelihood of anticipating future pet-related barriers to healthcare (see Table 4).

### Moderation models

**Fig 3:**
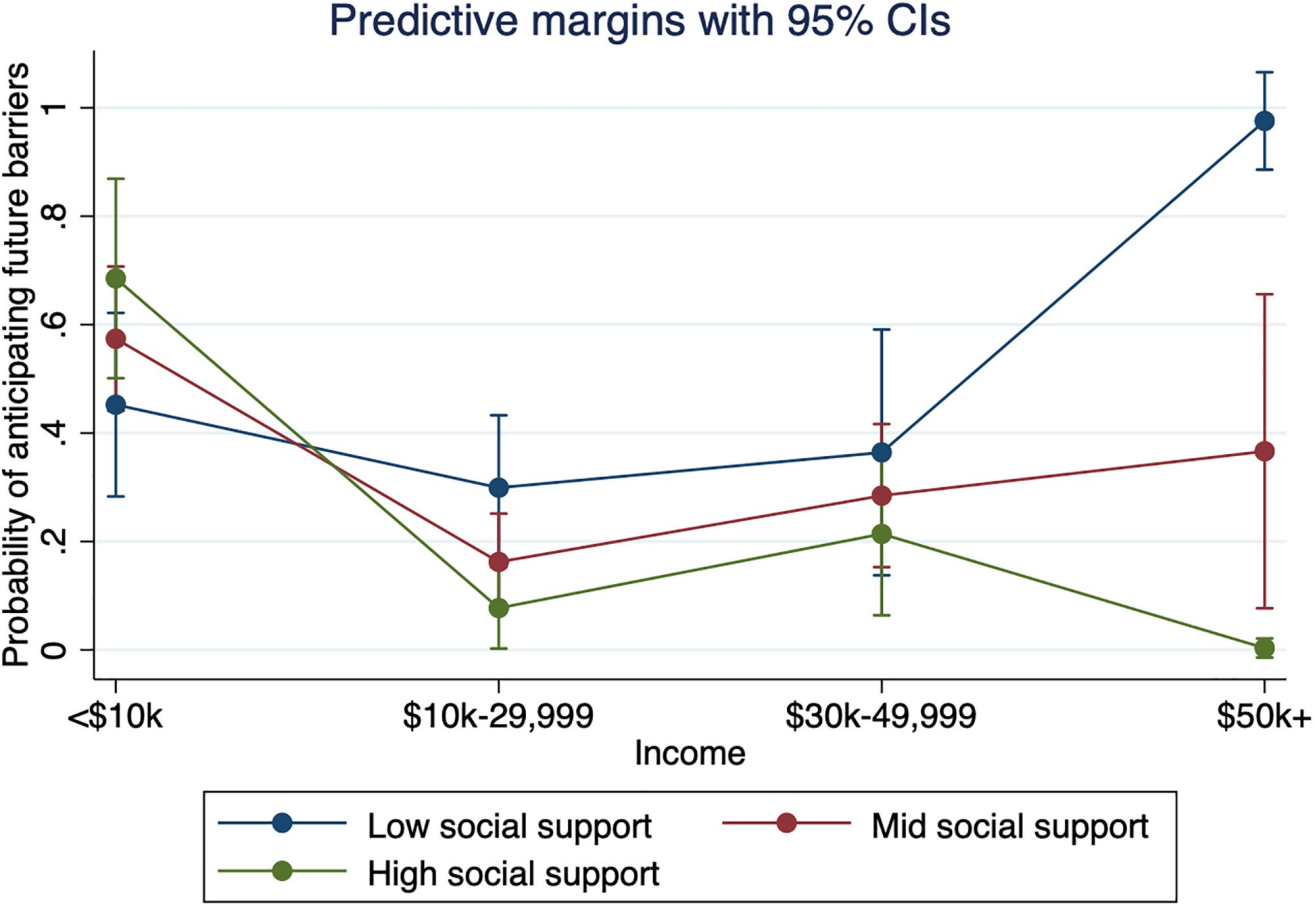
Social support moderates the effect of income on the probability of anticipating future pet-related barriers to healthcare (*n* = 204). Adjusted for race, Hispanic ethnicity, comfort from companion animals, pet type, number of pets, and healthcare needs.

Social support did not significantly moderate the effect of income on the likelihood of having experienced previous pet-related barriers to healthcare. However, social support did significantly moderate the effect of income on the likelihood of anticipating future healthcare barriers. Those with low social support scores were more likely to anticipate future healthcare barriers than those with mid or high social support scores at all income levels except <$10,000 per year. As can be observed in Fig 3, among those who made $50,000 per year and above, those with low social support scores were substantially more likely than those with mid or high social support scores to anticipate future healthcare barriers (*OR* = 0.61, *p* = 0.015).

The interactions between social support and comfort from companion animals were non-significant in all models, suggesting no evidence of moderation.

## Discussion

In this study, we sought to gain a greater understanding of how pet ownership may impact healthcare utilization and access for PWH. We hypothesized that lower levels of social support, greater comfort from pets, and lower income would be associated with more pet-related barriers to healthcare. Additionally, we hypothesized that social support would moderate the effect of both income and comfort from pets on pet-related barriers to healthcare. We found that over one-third of the participants endorsed at least one item on the pet-related barriers to healthcare index, and comparably more anticipated future healthcare barriers than those who had previously actually experienced them. Income, social support, comfort derived from companion animals, whether the participant was taking ART, and race were important factors in predicting the extent to which participants experienced or anticipated healthcare barriers.

Prior research has shown that income can be a salient factor in the ability of PWH and pet owners to access healthcare [20, 33]. Our findings regarding income were mostly consistent with prior research: those with greater economic resources had fewer total healthcare barriers and were also less likely to anticipate experiencing future healthcare barriers than those with lower household incomes. However, those with greater income were more likely to report previously experienced healthcare barriers than those with lower income. We suspect that this conflicts with prior studies of pet owners due to the comparably low average income in this sample compared to previous studies. For example, while those reporting incomes at $50,000 are likely better able to maintain basic needs than those reporting incomes under $10,000, they still may not be able to afford expensive pet-related services such as pet sitting or boarding. It is important to note that, due to data limitations, we did not consider the number of people in the household who are being supported by the total income reported, nor did we consider the cost-of-living and built environmental differences by location, which can vary considerably across the state of Florida. An alternative explanation could be that the participants with more financial resources may have connected their pets with more veterinary resources, therefore identifying more pet health and related resource needs. Relatedly, there may be a service gap for middle-income earners who cannot access subsidized services for both themselves and their pets, thus making pet-related barriers to healthcare a more salient problem, even for those who are not considered low-income. All of these factors should be considered in future research that investigates the role of household income in pet-related barriers to healthcare access.

Social support was an important factor across all models. This is consistent with previous research on this topic, which consistently shows that those who have greater access to social support are better able to care for their own health needs without concern for their pets’ welfare [33, 35]. We also found that social support moderated the effect of income on the likelihood of anticipating future pet-related barriers to healthcare, and the moderating impact of social support was most salient for those making $50,000+. Among this income group, those with low social support had a significantly higher probability of anticipating future healthcare barriers than those with mid or high social support. While social support is likely very important in low-income communities for other reasons, such as emotional support, the limitations of broader network socioeconomic status may prevent them from the provision of instrumental support [48], such as contingency care for pets. Additionally, those in households making income above the threshold for supplemental social and economic services may depend more heavily upon social support from their network than those who receive government and community support for things like subsidized housing and access to food banks, as well as pet services. For example, many communities offer free or low-cost veterinary services for very low-income individuals, while those making over a certain threshold would not qualify for the same services [49].

Emotional comfort from pets also influenced the extent to which participants reported pet-related barriers to healthcare: those who derived more comfort from pets were more likely to anticipate future barriers. Although a strong attachment bond to a pet is thought to facilitate health and well-being among PWH, some research has indicated that support from a pet may not be directly related to improved well-being when human social support is insufficient [50, 51]. While we did not find evidence for an interaction between human social support and comfort from pets on the likelihood of having experienced previous barriers nor anticipating future barriers, future research with a larger sample size should consider the moderating role of human social support on the relationship between comfort from pets and healthcare access and utilization.

Race was significantly associated with the likelihood of anticipating future pet-related barriers to healthcare: those who identified their race as Black were less likely than White participants to anticipate future healthcare barriers. Previous research has shown racial disparities in access to healthcare (e.g., [52, 53]) and broader health disparities as a consequence of racism and marginalization (e.g., [54–57]); thus, we would have anticipated that Black participants would have greater pet-related barriers to healthcare than others. Given the dearth of research on the relationship between race and relationships with pets, an important future area of research is the impact of racism and concomitant social and structural factors on pet-related barriers to healthcare and other pet-related health outcomes.

Due to the importance of regular health maintenance and consistent engagement with the healthcare system, any potential barrier to care could be a threat to health for PWH [20]. To this end, we found that participants who were taking ART were more likely to report a greater number of barriers than those who were not, indicating that pet-related barriers to healthcare are likely a more salient concern for those taking steps to actively manage the disease. We did not find significant associations between other indicators of healthcare needs and pet-related barriers to care; however, this could be due to our sample size. It is possible that a study with a larger sample and thus more power could detect other significant effects related to healthcare needs and the complexity of one’s care plan.

Prior research on the salience of support from pets for this population indicates that it is pertinent that PWH can access the care they need without compromising the human-animal bond. This study did not investigate whether pet-related barriers to healthcare were associated with poorer HIV health outcomes (i.e., viral load, CD4 count); future research should consider whether these healthcare barriers impact health outcomes among PWH. Furthermore, it is likely that this issue is salient among individuals with other chronic diseases, such as cancer, heart disease, or diabetes. Future research should consider this issue of pet-related barriers to healthcare among people with other chronic diseases.

We strongly caution against any interpretation of this study that could be misconstrued to assume that, because pets may be a barrier to healthcare for some PWH, the solution is to take away or prevent them from acquiring pets. We contend that the solutions to these issues of healthcare access and poor health are best addressed in higher-level public health interventions that harness the human-animal bond, rather than deny or ignore it.

## Conclusion

In this study, we found that a notable proportion of PWH reported experiencing or anticipating pet-related healthcare barriers, such as delaying care or leaving in-patient treatment to care for pets. Social support, comfort derived from pets, income, race, and ART status were associated with experiences of pet-related barriers to healthcare. We recommend community support for the pets of those managing chronic diseases, such as HIV, to better facilitate access to healthcare without compromising the human-animal bond. For example, to increase access to family healthcare and veterinary resources, communities may consider partnerships between animal welfare organizations and community health clinics [58]. If PWH who have pets find they have trouble maintaining their medication regimen or healthcare maintenance, healthcare providers may consider a solution that allows patients to bring their pets to appointments with them. Ideally, community health clinics would even partner with veterinarians to offer free or subsidized veterinary care to low-income pet owners. Additionally, providing temporary boarding or fostering services could alleviate problems with longer-term healthcare needs. The provision of pet food and supplies at food banks that serve low-income pet owners could also make strides in removing barriers to one’s own health maintenance.

## Supporting information

S1 Checklist STROBE statement—Checklist of items that should be included in reports of observational studies. (DOCX)
