# Correction: The impact of pet ownership on healthcare access and utilization among people with HIV

**DOI:** 10.1371/journal.pone.0299559

**Published:** 2024-02-23

**Authors:** Jennifer W. Applebaum, Shelby E. McDonald, Maya Widmeyer, Humberto E. Fabelo, Robert L. Cook

## Notice of republication

This article was republished on February 13, 2024, to correct the error in the abstract. Irrelevant citations were included in the abstract. Please download this article again to view the correct version. The originally published, uncorrected article and the republished, corrected articles are provided here for reference.

## Supporting information

S1 File Originally published, uncorrected article. (PDF)

S2 File Republished, corrected article. (PDF)
