# Supplementary material for: The impact of pet ownership on healthcare access and utilization among people with HIV
Source: PLoS One. 2023 Nov 1;18(11):e0292658. doi: 10.1371/journal.pone.0292658 (PMC10619778; doi:10.1371/journal.pone.0292658)
Supplement: S1 Checklist — (DOCX) [file pone.0292658.s001.docx]

STROBE Statement—checklist of items that should be included in reports of observational studies

|  | Item No. | Recommendation | Page  No. | Relevant text from manuscript |
| --- | --- | --- | --- | --- |
| **Title and abstract** | 1 | (*a*) Indicate the study’s design with a commonly used term in the title or the abstract | 2 | Pet-owning PWH (*n*=197) were recruited at healthcare and community sites throughout Florida as part of a larger survey study (the “Florida Cohort”). |
|  |  | (*b*) Provide in the abstract an informative and balanced summary of what was done and what was found | 2 | We developed a 12-item index of pet-related barriers to healthcare, which was designed to assess whether the participants experienced or anticipated any barriers to accessing and/or utilizing timely healthcare or health-related services due to pet caregiving or concerns about pet welfare. We estimated a series of regression models (negative binomial, logistic regression) to assess the effects of comfort from companion animals, human social support, and sociodemographic characteristics on (1) the total number of pet-related healthcare barriers endorsed, (2) previously experienced pet-related healthcare barriers, and (3) anticipated pet-related healthcare barriers. Thirty-six percent of the sample reported at least one experienced or anticipated pet-related barrier to their healthcare; 18% reported previous healthcare barriers and 31% anticipated future healthcare barriers. Greater comfort from companion animals and poorer social support were associated with a greater probability of experiencing or anticipating any pet-related healthcare barriers. Those who identified racially as Black were less likely to anticipate future healthcare barriers than those who were White. Income was associated with healthcare barriers in all models. |
| Introduction | | | |  |
| Background/rationale | 2 | Explain the scientific background and rationale for the investigation being reported | 3-7 | See Introduction |
| Objectives | 3 | State specific objectives, including any prespecified hypotheses | 7-8 | See Current Study |
| Methods | | | |  |
| Study design | 4 | Present key elements of study design early in the paper | 8-9 | See Methods |
| Setting | 5 | Describe the setting, locations, and relevant dates, including periods of recruitment, exposure, follow-up, and data collection | 8-9 | See Methods |
| Participants | 6 | (*a*) *Cohort study*—Give the eligibility criteria, and the sources and methods of selection of participants. Describe methods of follow-up  *Case-control study*—Give the eligibility criteria, and the sources and methods of case ascertainment and control selection. Give the rationale for the choice of cases and controls  *Cross-sectional study*—Give the eligibility criteria, and the sources and methods of selection of participants | 8-9 | See Methods |
|  |  | (*b*) *Cohort study*—For matched studies, give matching criteria and number of exposed and unexposed  *Case-control study*—For matched studies, give matching criteria and the number of controls per case |  |  |
| Variables | 7 | Clearly define all outcomes, exposures, predictors, potential confounders, and effect modifiers. Give diagnostic criteria, if applicable | 10-13 | See Measures |
| Data sources/ measurement | 8* | For each variable of interest, give sources of data and details of methods of assessment (measurement). Describe comparability of assessment methods if there is more than one group | 10-13 | See Measures |
| Bias | 9 | Describe any efforts to address potential sources of bias | 9, 14 | See Sample Characteristics, Analytic Procedures |
| Study size | 10 | Explain how the study size was arrived at | 8-9 | See Methods |

Continued on next page

| Quantitative variables | 11 | Explain how quantitative variables were handled in the analyses. If applicable, describe which groupings were chosen and why | 14 | See Analytic Procedures |
| --- | --- | --- | --- | --- |
| Statistical methods | 12 | (*a*) Describe all statistical methods, including those used to control for confounding | 14 | See Analytic Procedures |
|  |  | (*b*) Describe any methods used to examine subgroups and interactions | 14 | See Analytic Procedures |
|  |  | (*c*) Explain how missing data were addressed | 14 | See Analytic Procedures |
|  |  | (*d*) *Cohort study*—If applicable, explain how loss to follow-up was addressed  *Case-control study*—If applicable, explain how matching of cases and controls was addressed  *Cross-sectional study*—If applicable, describe analytical methods taking account of sampling strategy | N/A |  |
|  |  | (*e*) Describe any sensitivity analyses | N/A |  |
| Results | | | | |
| Participants | 13* | (a) Report numbers of individuals at each stage of study—eg numbers potentially eligible, examined for eligibility, confirmed eligible, included in the study, completing follow-up, and analysed | 9 | See Sample Characteristics |
|  |  | (b) Give reasons for non-participation at each stage | N/A |  |
|  |  | (c) Consider use of a flow diagram |  |  |
| Descriptive data | 14* | (a) Give characteristics of study participants (eg demographic, clinical, social) and information on exposures and potential confounders | 9 | See Sample Characteristics |
|  |  | (b) Indicate number of participants with missing data for each variable of interest | 9 | See Sample Characteristics |
|  |  | (c) *Cohort study*—Summarise follow-up time (eg, average and total amount) | N/A |  |
| Outcome data | 15* | *Cohort study*—Report numbers of outcome events or summary measures over time | N/A |  |
|  |  | *Case-control study—*Report numbers in each exposure category, or summary measures of exposure | N/A |  |
|  |  | *Cross-sectional study—*Report numbers of outcome events or summary measures | 10, 14-15 | See Table 1, Figure 1, and Descriptive information |
| Main results | 16 | (*a*) Give unadjusted estimates and, if applicable, confounder-adjusted estimates and their precision (eg, 95% confidence interval). Make clear which confounders were adjusted for and why they were included | 15-18 | See Results |
|  |  | (*b*) Report category boundaries when continuous variables were categorized | N/A |  |
|  |  | (*c*) If relevant, consider translating estimates of relative risk into absolute risk for a meaningful time period | N/A |  |

Continued on next page

| Other analyses | 17 | Report other analyses done—eg analyses of subgroups and interactions, and sensitivity analyses | 14 | See Analytic Procedures |
| --- | --- | --- | --- | --- |
| Discussion | | | | |
| Key results | 18 | Summarise key results with reference to study objectives | 18-22 | See Discussion |
| Limitations | 19 | Discuss limitations of the study, taking into account sources of potential bias or imprecision. Discuss both direction and magnitude of any potential bias | 18-22 | See Discussion |
| Interpretation | 20 | Give a cautious overall interpretation of results considering objectives, limitations, multiplicity of analyses, results from similar studies, and other relevant evidence | 18-22 | See Discussion |
| Generalisability | 21 | Discuss the generalisability (external validity) of the study results | 18-22 | See Discussion |
| Other information | |  | | |
| Funding | 22 | Give the source of funding and the role of the funders for the present study and, if applicable, for the original study on which the present article is based | 23 | See Funding and Acknowledgments |

*Give information separately for cases and controls in case-control studies and, if applicable, for exposed and unexposed groups in cohort and cross-sectional studies.

**Note:** An Explanation and Elaboration article discusses each checklist item and gives methodological background and published examples of transparent reporting. The STROBE checklist is best used in conjunction with this article (freely available on the Web sites of PLoS Medicine at http://www.plosmedicine.org/, Annals of Internal Medicine at http://www.annals.org/, and Epidemiology at http://www.epidem.com/). Information on the STROBE Initiative is available at www.strobe-statement.org.
